# Eco-Morphological Responses of *Camponotus japonicus* (Hymenoptera: Formicidae) to Varied Climates and Habitats

**DOI:** 10.3390/insects15090719

**Published:** 2024-09-19

**Authors:** Ruoqing Ma, Liangliang Zhang, Hong He

**Affiliations:** Key Laboratory of National Forestry and Grassland Administration for Control of Forest Biological Disasters in Western China, College of Forestry, Northwest A&F University, Xianyang 712100, China

**Keywords:** ant, climate, habitat, mainland China, morphological traits

## Abstract

**Simple Summary:**

Ants are highly adaptable insects that thrive in a variety of climates and habitats worldwide. This study examines how climate and habitat influence the morphological traits of the ant species *Camponotus japonicus* across 22 sites in mainland China. These sites represent three climate zones and three habitat types. Our analysis shows that both climate and habitat significantly shape the ants’ morphological traits. Specifically, ants in mid-temperate farmlands exhibit more constrained morphological traits, while those in sparse woodlands show greater variation. Urban parks present a stable environment with less morphological variation. Temperature, precipitation, humidity, and altitude were found to be closely linked to these morphological traits. This research enhances our understanding of how ants adapt to environmental changes through morphological variation and underscores their critical ecological roles in various ecosystems.

**Abstract:**

Ants are a highly adaptable group of insects that have globally established themselves in diverse climates and habitats. This study investigates the influence of climate and habitat on the morphological traits of *Camponotus japonicus* across 22 sites in mainland China. These sites span three climate zones (mid-temperate, warm temperate, and subtropical) and three habitat types (urban parks, farmlands, and sparse woodlands). Principal component analysis (PCA) was used to determine the principal axis of morphological variation, while hypervolume analysis and centroid distance calculation were used to verify the environmental filtering hypothesis and the optimal transfer hypothesis. The results support both hypotheses showing that climate and habitat significantly affect the morphological space of *C. japonicus* workers. In particular, the morphological space is more constrained in mid-temperate farmlands, while workers in sparse woodlands exhibit greater morphological variation. In contrast, urban parks are characterized by higher stability and reduced morphological differences. Additionally, robust regression analysis reveals that environmental factors such as temperature, precipitation, humidity, and altitude are closely linked to the morphological traits of the workers. Understanding how ant morphology responds to external environmental changes enhances our understanding of their adaptability and their essential ecological roles across various ecosystems.

## 1. Introduction

The study of morphological variation in organisms in response to climate and environmental changes has long been a challenge in ecological research [1,2,3,4,5,6]. Classic eco-geographical rules, such as Bergmann’s rule, Gloger’s rule, and Allen’s rule, provide frameworks to explain how morphological traits in both warm-blooded and cold-blooded animals adapt to external environmental factors [1,2,7]. These morphological changes can also result from phenotypic plasticity, where a single genotype exhibits multiple phenotypes in response to different environments [8,9,10]. Adaptive responses are shaped by directional selection, which favors plastic responses that optimize fitness in changing environments [11]. In this context, the environmental filtering hypothesis and the optimal transfer hypothesis provide a key theoretical framework for our understanding of species morphological change. The environmental filtering hypothesis holds that in more severe or resource-limited environments, strong selection pressure will lead to the convergence of morphological characteristics within a group, narrowing its morphological space [12,13]. The optimal transfer hypothesis proposes that different environments may support different optimal phenotypes, so that populations shift in morphological space rather than simply shrink [12,13,14].

The direct ramifications impact of climate change encompasses a rise in temperature, modifications to precipitation patterns, and an escalation in the frequency and intensity of extreme weather events [15,16]. These changes profoundly impact biological habitats, altering the distribution and abundance of species, disrupting food webs and ecosystem processes, and increasing the risk of extinction for vulnerable species [17,18]. At the same time, habitat changes, such as farmland reclamation, deforestation, and urbanization, further increase the adaptive pressure on species [19,20]. The concurrent occurrence of climate warming and habitat alteration will inevitably increase the cost of biological adaptation. Consequently, studying the adaptation mechanism of species to these environmental changes, especially the changes in morphological traits, is one of the intuitive ways to understand species’ response to environmental selection. In addition, the role of intraspecific trait variation (ITV) in community ecology and ecosystem function has received increasing attention in recent years [21]. Intraspecific trait variation refers to the differences in traits of individuals within the same species, which can not only affect individual adaptability, but also change the interaction, competition, and resource allocation among species at the community level [22]. Studies have shown that intraspecific trait variation can affect ecosystem function through multiple mechanisms, including enhancing the competitive advantage of species in a community, increasing the niche width of a community, and promoting ecosystem stability [23]. In ecosystems with limited resources or high environmental pressure, intraspecific trait variation may enhance the buffering capacity of communities to environmental changes, thereby maintaining the stability of ecosystem functions [24].

Species with wide geographical distribution and high ecological plasticity levels can survive in a variety of climatic conditions and habitat types [15]. Insects represent a taxonomically diverse group of organisms that exhibit remarkable phenotypic plasticity in a range of traits, including morphology, pigmentation, and behavioral traits [25,26,27]. Among them, ants are one of the most common and highly evolved social insects in nature, distributed on all continents except Antarctica [28,29] and dominate terrestrial ecosystems in terms of quantity and biomass [30,31]. Their ecological importance as soil architects, seed dispersers, and predators makes them critical components of many ecosystems [32,33]. As eusocial insects, the sociality of ants provides them with behavioral and phenotypic plasticity to cope with diverse environmental conditions [16]. The presence of non-reproductive division of labor is a salient feature of eusocial insects, in which specialized and sexually sterile worker castes operate together with the reproductive caste of the queen to produce the reproductive division of labor [34,35]. The worker caste, known for its highly organized and cooperative behavior, constitutes the largest demographic within the colony and performs a key role in ensuring the survival of the colony [30,36]. They are the primary foragers and builders of the colony, responsible for tasks such as gathering food, caring for the queen and young brood, and constructing the nest [37]. In many ant species, their workers can be divided into “major workers” and “minor workers”, which are clearly different in body sizes and duties [38,39]. Moreover, some ant populations demonstrate significant adaptability by altering their morphology, body size, and behavioral strategies in response to the micro-ecological environments [40,41]. For example, *Camponotus crassus* exhibits flexible foraging and defense behaviors, adapting to seasonal changes in food supply and environmental changes [42]. In seasonal environments, ants such as *Pogonomyrmex naegelii* adjust their diet as the wet and dry seasons change, from a seed-based diet to a diet that also includes meat [43]. In addition, the foraging strategies and social behavior of *Camponotus senex* reveal the complexity of its community structure and division of labor. Studies have shown that these workers are not only involved in foraging but also exhibit advanced nest building techniques, demonstrating their adaptability to biotic and abiotic stresses [44]. This high sensitivity to environmental changes makes ants valuable ecological indicators across various ecological contexts [45].

Our studied organism, *Camponotus japonicus*, is a widely distributed ant species in mainland China [46]. It not only has relatively large body size and colonies but also has obvious polymorphism [47,48]. At the same time, this ant has a significant predatory effect on the larvae of *Dendrolimus punctatus*, effectively controlling the occurrence of these pests [49]. As a species with a wide distribution in regions with complex climatic conditions, its full ecological indicator potential has not been fully realized. Similar to some ant species that are widely distributed in tropical areas, *C. japonicus* also shows high ecological flexibility and strong environmental adaptability in morphological characteristics [42]. By studying the variation in morphological traits of workers of *C*. *japonicus* across different climate zones and habitat types, we tested how isolated populations occupy morphological space and whether these differences are influenced by the strength of environmental filtering (environmental filtering hypothesis). Three different climate zones and three different habitat types were selected, resulting in nine unique environmental combinations. Most studies use the mean of morphological traits to represent the entire species. However, this approach ignores the variation in intraspecific traits and may underestimate the competitive ability of the species, as well as the extent of overlap between its niches and traits [50,51]. Therefore, by using eight morphological traits for principal component analysis, the first four principal components with the largest total variance explanation were extracted to represent the overall morphological changes. If the optimal phenotypic values in different environmental combinations drive directional selection and change the position of populations in morphological space, the strength of environmental filtering hypothesis is supported. On the other hand, if the range of ecological variation supported by the environmental combination is different (optimal transfer hypothesis), the volume of ecological space occupied by each ant population will be limited. At the same time, robust regression analysis was used to explore the relationships between environmental factors (such as temperature, precipitation, humidity, and altitude) and morphological traits. These analyses help to understand how ants interact with the environment and respond to habitat changes can also enhance our understanding of ecosystem functions and the impact of human activities on ecosystems.

## 2. Materials and Methods

### 2.1. Study Sites

**Figure 1:**
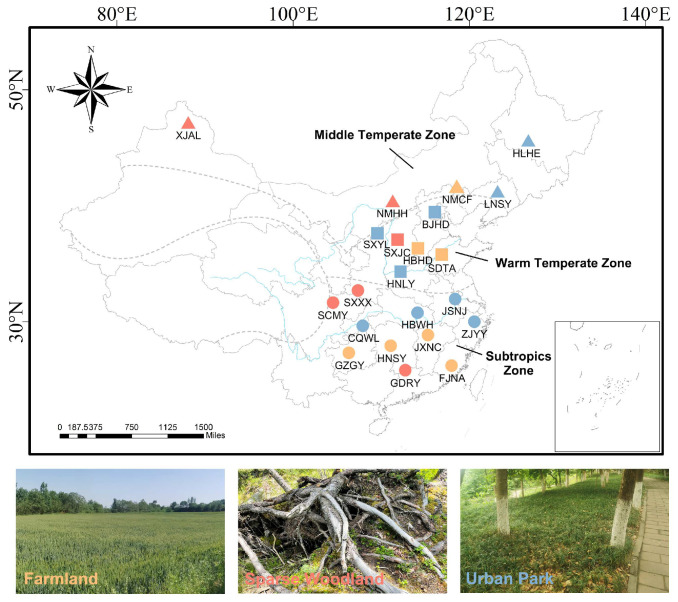
The geographic distribution and habitat types of *Camponotus japonicus* sampled in this study. The diagram incorporates a color-coded key to denote three distinct habitat types. Blue signifies urban parks, yellow represents farmlands, and red is allocated for sparse woodlands. Triangles demarcate the middle temperate zone, squares delineate the warm temperate zone, and circles indicate the subtropical zone.

This study was conducted in 22 sites that encompassed nearly all the distribution area of *C. japonicus* in mainland China, spanning three climate zones (middle temperate, warm temperate, and subtropical zones) and three habitat types (farmlands, sparse woodlands, and urban parks) (Table 1, Figure 1). The sampling sites were characterized by a range of environmental conditions, with mean temperatures varying from −0.65 °C to 21.52 °C and observed minimum and maximum temperatures of −18.89 °C and 28.71 °C. The mean precipitation ranged from 27.75 mm to 191.35 mm, with minimum and maximum values of 0.61 mm and 521.71 mm and altitudes from 35 m to 1275 m. The longitudinal coordinates of these sites extended from 88° to 126° east and latitudinal coordinates from 24° to 47° north. The climate zoning data were acquired from the Resource and Environmental Science and Data Center, which is affiliated with the Institute of Geographic Sciences and Natural Resources Research at the Chinese Academy of Sciences. Habitat classification is mainly based on land use classification. Farmlands are characterized by extensive cultivated fields and substantial crop coverage. Urban parks are man-made public green spaces within cities, featuring high green coverage. Sparse woodlands are areas within forested regions where tree coverage exists but is discontinuous, resulting in lower overall vegetation density.

### 2.2. Ant Sampling and Identification

From April to August 2021, the foraging workers (both major and minor) of *C. japonicus* were collected from 22 sites in mainland China. Three colonies were sampled at each site, ensuring an inter-nest distance of 80 to 100 m to prevent foraging area overlap. To minimize potential biases related to colony size, only colonies with more than 30 foraging workers and more than 10 major workers observed around the colony entrance were selected. Collections were conducted from 11 AM to 5 PM during clear weather conditions, with sampling occurring hourly within a 30 m radius around the nest entrances. This method, focused on actively foraging ants, was chosen to explore the influence of environmental selection on ant morphology under various climatic and habitat conditions.

The workers were sampled by hand-collecting as many as possible. All workers were stored in 100% ethanol and brought back to a laboratory for identification. The workers of *C. japonicus* were then identified based on taxonomic keys for ant fauna [52] and for the genus *Camponotus* [53]. One ant from each of the three colonies of each geographic population was selected to represent the entire colony for molecular identification. Genomic DNA was extracted using the BioTeke DNA extraction kit (Beijing, China), and PCR amplification of mitochondrial cytochrome oxidase enzyme I (*COI*) fragments was performed using the method described by Vrijenhoek [54]. Sequencing was conducted by Tsingke (Xi’an, China) Biotechnology Co., Ltd. All the sequences have been submitted to GenBank (Accession Numbers: PQ273030-PQ273095). The *COI* gene fragments of *Camponotus herculeanus* (MZ608010.1) and *Camponotus pennsylvanicus* (HM395022.1) were downloaded from the NCBI database and used as outgroups for analysis. Multiple sequence alignment was performed using MAFFT v.7.0 [55]. ModelFinder [56] was used to select the best-fit model using BIC criterion. Bayesian inference phylogenies were inferred using MrBayes 3.2.6 [57] under the HKY + G + F model (2 parallel runs, 2,000,000 generations), in which the initial 25% of sampled data were discarded as burn-in. Figtree v.1.4.4 software was used to visualize the topology [58]. Voucher specimens were deposited at the College of Forestry, Northwest A&F University (NWAFU), Yangling, Shaanxi Province, China.

### 2.3. Measurements of Ant Morphological Traits

Morphological traits were measured using an Olympus SZX10 microscope (Olympus Corporation, Tokyo, Japan) in conjunction with an SX60HS digital camera (Olympus Corporation, Tokyo, Japan). In each geographic population, 21 major and minor workers were selected for morphological measurements, with 7 individuals sampled from each of the three colonies per population. According to Zhang’s research [47] on the apparent dimorphism of *C. japonicus* workers in different geographical populations, the size of workers can be distinguished by the differences in body length and head morphology: the head of minor workers is oval- or barrel-shaped, while that of major workers is heart-shaped. Eight continuous morphological traits (Table S1) of functional significance were measured for each worker. These raw data were standardized and subsequently utilized in a principal component analysis employing varimax rotation to identify principal axes of morphological variation. The subsequent analyses retained the first 4 principal components (PCs), which accounted for a significant proportion of the total variance for major and minor workers. PCs 1 to 4 were strongly influenced by traits associated with body size, chemical sensory capacities, predatory strategies, defense strategies, and habitat exploration capabilities (Table S1).

### 2.4. Data Analysis

The first 4 PCs were used to analyze the morphological space of major and minor workers. In each geographic population, 10 workers (major and minor workers were sampled separately) were randomly selected, with at least 3 individuals from each colony to reduce potential morphological differences between colonies. The “hypervolume” package in R ver. 4.1.1 [59] was used to calculate the convex hull, and this process was repeated 100 times. To generate a random expectation and evaluate significance, 10 individuals from all individuals (major and minor workers sampled separately) were randomly selected, and this process was repeated 1000 times. To test the optimal transfer hypothesis, the distance from each population’s morphological space centroid to the centroid of all pooled populations was calculated. This distance was compared to a random expectation using 1000 simulations. Specifically, for each simulation, 10 individuals were randomly sampled from each population, with at least 3 individuals from each colony. The distance between this sample centroid and the centroid of all populations pooled was then computed. Given the non-normal distribution of the random expectation, Mann–Whitney U test was employed to assess differences in convex hull volume and centroid distance between populations (i.e., the distance of population centroids in morphological space from the overall centroid). For all analyses, the significance was set at *p* < 0.001.

The Scheirer–Ray–Hare test was conducted for each principal component value, using the “scheirerRayHare” function from the “rcompanion” package in R. This analysis allowed for the assessment of the effects of the climate and habitat as well as their interaction. Prior to the variance analysis, the assumptions of normality and homogeneity of variances were verified. To reduce the risk of type I errors due to multiple comparisons of PC values, Bonferroni correction was applied to the *p*-values to ensure robust significance and prevent false positives. The significance was set at *p* < 0.001.

Robust regression was performed using the “rlm” function from the “MASS” package to examine the relationship between the first 4 PC values and 4 environmental factors (annual temperature, annual mean precipitation, relative humidity, and elevation). The environmental factors data were obtained from National Centers for Environmental Information (NCEI), NOAA (Table S2).

## 3. Results

A total of 880 workers in 22 sampling sites were measured, including 440 major and minor workers each. The phylogenetic tree constructed using Bayesian inference provides robust support for the monophyly of various *C. japonicus* populations (Bayesian posterior probability is 1.0) (Figure S1). Both the identification key and phylogenetic analysis consistently confirm that the collected ants belong to *C. japonicus*.

**Figure 2:**
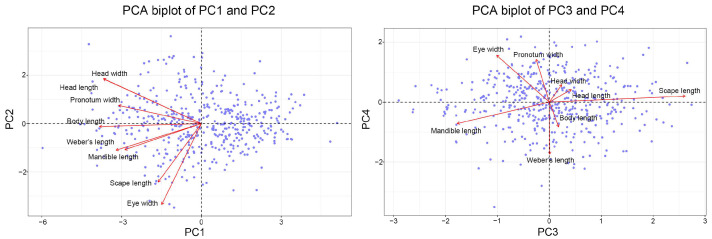
Principal component analysis (PCA) biplots of major worker morphological traits: PC1 vs. PC2 and PC3 vs. PC4.

**Figure 3:**
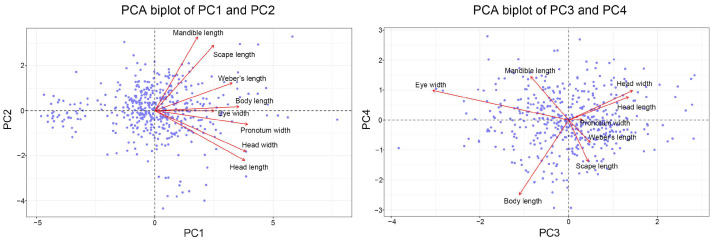
Principal component analysis (PCA) biplots of minor worker morphological traits: PC1 vs. PC2 and PC3 vs. PC4.

The first four PCs of major workers explained 76.98% of the total variance, with PC1 explaining 41.73% of the variance, mainly reflecting variations in head size and pronotum width. PC2 explained 14.97% of the variance, indicating variations in scape and Weber’s length. PC3 explained 11.17% of the variance and highlighted variations in mandible length. PC4 explained 9.11% of the variance and reflected additional variations in eye width (Table 2, Figure 2). Similarly, the first four PCs of minor workers explained 74.99% of the total variance. PC1 explained 39.40% of the variance, primarily reflecting variations in head size and pronotum width. PC2 explained 13.78% of the variance, indicating variations in scape and mandible length. PC3 explained 11.82% of the variance, highlighting variations in eye width. PC4 explained 9.99% of the variance and reflected variations in mandible length (Table 3, Figure 3).

**Figure 4:**
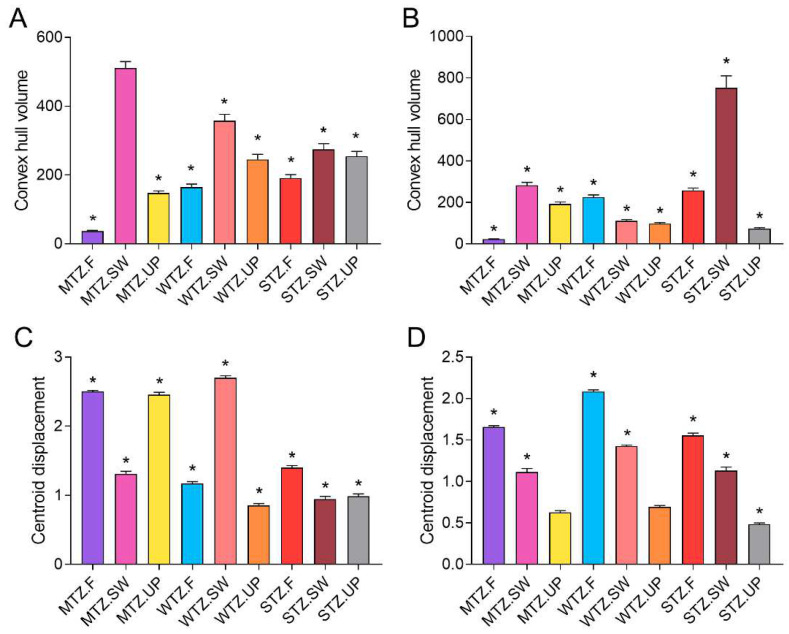
Morphological space (convex hull) volume and centroid displacement for major and minor workers across nine climate–habitat types. (**A**) Convex hull volume for major workers. (**B**) Convex hull volume for minor workers. (**C**) Centroid distances for major workers. (**D**) Centroid distances for minor workers. An asterisk (*) indicates a *p*-value less than 0.001 based on the random model. The convex hull volume represents the degree of clustering of traits. The centroid displacement represents the distance between the centroid of the morphological space of each population and the centroid of all populations.

Morphological space (convex hull) volume clustering was significant among the nine composite climate–habitat types for major and minor workers, except MTZ.SW. For major workers, the convex hull volume of MTZ.F is smaller than that of other climate–habitat types, while the convex hull volume of MTZ.SW and WTZ.UP is larger (Figure 4A). Similarly, for minor workers, the convex hull volume of MTZ.F is also significantly smaller than that of other climate–habitat types, and that of STZ.SW is higher than that of others (Figure 4B). The centroid distance of major and minor workers is significantly higher than the random expectation value, except for MTZ.UP and WTZ.UP. For major workers, MTZ.F, MTZ.UP, and WTZ.SW were larger than other climate–habitat types (Figure 4C). For minor workers, MTZ.UP, WTZ.UP, and STZ.UP are significantly lower than others (Figure 4D).

The Scheirer–Ray–Hare test revealed significant effects of climate zones, habitat types, and their interaction on the morphological traits of major and minor workers. For PC1, the effects of climate zones (H = 144.78, *p* < 0.001, Bonferroni-corrected *p* < 0.001) and their interaction with habitat types (H = 50.04, *p* < 0.001, Bonferroni-corrected *p* < 0.001) were significant. Similarly, for PC2, the effect of habitat types (H = 31.05, *p* < 0.001, Bonferroni-corrected *p* < 0.001) and their interaction with climate zones (H = 59.42, *p* < 0.001, Bonferroni-corrected *p* < 0.001) were significant. For PC3 and PC4, the interaction between climate zones and habitat types were significant (PC3: H = 43.76, *p* < 0.001, Bonferroni-corrected *p* < 0.001; PC4: H = 36.07, *p* < 0.001, Bonferroni-corrected *p* < 0.001) (Table 4). For minor workers, the effects of climate zones (H = 29.95, *p* < 0.001, Bonferroni-corrected *p* < 0.001) and their interaction with habitat types (H = 67.03, *p* < 0.001, Bonferroni-corrected *p* < 0.001) were significant for PC1. Similarly, for PC3, the interaction between climate zones and habitat types were significant (H = 93.14, *p* < 0.001, Bonferroni-corrected *p* < 0.001). For PC2 and PC4, the effects of climate zones, habitat types, and their interaction were significant for all comparisons (*p* < 0.001, Bonferroni-corrected *p* < 0.001) (Table 5).

Based on the robust regression results of major workers, the effects of environmental factors on principal components values are different (Table 6). The annual mean temperature had a significant positive correlation with PC1 and PC2 but had no significant effect on PC3 and PC4. The average annual precipitation has a significant positive correlation with PC1 and PC3, and a significant negative correlation with PC4. Relative humidity has a significant positive effect on PC4 but a weak effect on other principal components. Altitude has a significant negative correlation with PC1 and PC3 but no significant effect on PC2 and PC4. Similarly, for minor workers, the annual mean temperature has a significant negative effect on PC1, PC2, and PC3 but has no significant effect on PC4 (Table 7). The annual mean precipitation has a significant positive correlation with PC1, PC2, and PC3 but has no significant effect on PC4. Elevation has a significant negative effect on all principal component values.

## 4. Discussion

Climate and ecological environment significantly influence ant populations, affecting their distribution, morphology, and behavior [16,46,60]. This study systematically explored the impact of climate and habitat factors and their interactions on *C. japonicus* through large-scale sampling in three climate zones: mid-temperate, warm temperate, and subtropical, as well as three habitat types: urban parks, farmlands, and sparse woodlands, in mainland China.

In analyzing the morphological space volume, the major and minor workers in all the plots were significantly smaller than the random expectations. This indicates a significant clustering of morphological traits of *C. japonicus* under different environmental conditions, supporting the environmental filtering hypothesis [12]. The harshness of environmental conditions or resource limitations can cause convergence of morphological traits within a population, thereby reducing morphological diversity [12,14]. Climate change can further exacerbate this morphological convergence by affecting resource availability and habitat structure [14,43]. For example, higher temperatures may speed up metabolism and increase competitive pressure, thus affecting ant morphological development [16]. The morphological space volume of both major and minor workers in middle temperate farmlands (MTZ.F) was significantly smaller than that in other combinations. This suggests that the combined effects of lower temperatures in the middle temperate climate and uniform agricultural management practices on farmlands exert continuous environmental pressures, resulting in a more homogeneous habitat [61,62]. This environmental pressure promotes the clustering of morphological features and reduces morphological diversity. In contrast, high temperatures and abundant resources in subtropical regions promote the expansion of ant morphological space volume, which allows ants to adapt to the environment within a wider range of morphological variation and exhibit greater morphological diversity. Additionally, the morphological space volume of major workers in sparse woodlands across the three climatic zones was significantly higher than that in farmlands and urban parks, indicating that morphological variability is higher in sparse woodland environments. Sparse woodlands provide a variety of microhabitats and opportunities for resource utilization, thereby promoting greater biological and morphological diversity by allowing species to exploit different ecological niches [63,64]. In contrast, farmlands and urban parks are relatively homogeneous environments with more consistent resource utilization, leading to stronger environmental filtering that reduces morphological variability [14,65].

The centroid distance results support the optimal transfer hypothesis. The optimal transfer hypothesis holds that different morphological characteristics have different optimal values in different environments, which leads to the differentiation of morphological characteristics in different populations [12]. For minor workers, the centroid distances for all farmland and sparse woodland habitats were greater than urban parks, indicating significant morphological variations in minor workers within farmland and sparse woodland habitats. In contrast, the urban park environment appears relatively stable, exerting less influence on morphological traits. This is consistent with the fact that urban parks typically offer abundant resources and fewer environmental pressures, resulting in ant populations experiencing minimal selective pressure on their morphological traits [14,66]. In addition, in more challenging environments such as middle temperate farmlands, ants display more uniform morphological traits to accommodate singular environmental pressures, whereas in more diverse environments like warm temperate sparse woodlands, ants’ morphological traits exhibit greater plasticity to diverse environmental conditions.

Ants can adapt to their ecological surroundings by altering their shape, size, and behavioral strategies, with behaviors such as aggression, foraging, and brood care particularly pivotal in adapting to temperature variations [40,41,67]. The results of this study showed that the environmental factors had a significant impact on the morphological traits of *C. japonicus* workers, and the influence patterns of different environmental factors on the principal components (PC1–4) of morphological traits were different. Previous studies have shown that temperature and precipitation can have profound effects on the morphological traits of ants, influencing their size, shape, and overall fitness [46,68,69]. For the major workers of *C. japonicus*, temperature and precipitation were positively correlated with the principal components of morphological traits that mainly reflect body size and predation ability, indicating that the body size and predation ability of major workers may increase under high temperatures and humid environments. For minor workers, temperature was negatively correlated with PC, which mainly reflected body size and predatory ability, while precipitation was positively correlated with PC. Temperature has a significant effect on metabolism and the thermal tolerance of organisms, and ants living in tropical and warm microclimates may experience greater stress in the face of increased temperatures, resulting in morphological changes [16]. The high temperature promoted the metabolic rate of the major workers, thus accelerating the growth of body size. At the same time, the wet environment may have increased the available food resources, further promoting its size and predatory ability. Both intraspecific and interspecific competition may influence body size change through foraging behavior and prey selection [70]. Altitude is also a key environmental factor, often affecting the ecological and physiological characteristics of ants, resulting in changes in their body size and adaptation to environmental conditions [71,72]. It was found that the altitude was negatively correlated with the body size and predatory ability of major and minor workers, indicating that the high-altitude environment limited the size of ants. At cold, high altitudes, the environment does exert a limiting effect on the size and predatory ability of ants [73].

In conclusion, this study investigated the significant effects of climate and habitat on the morphological traits of *C. japonicus* workers in different ecological regions in mainland China. The results support the environmental filtering hypothesis, suggesting that harsher environmental conditions or resource constraints lead to the convergence of morphological traits, thereby reducing diversity within a population. In contrast, ant morphological diversity increased in resource-rich environments with diverse microhabitats, such as subtropical woodlands. The significant correlation between climate factors and the principal components of morphological traits further suggests that there is a complex interaction between climate and morphological change. This study deepens our understanding of how ants adapt to environmental stress through morphological changes and highlights the importance of considering climate and habitat when studying land insects. The study of the dynamic relationship between ants and their environment will help to deepen the understanding of the role of ants as ecological indicator species and provide a new perspective for predicting and coping with the ecological impact of environmental change.

## Figures and Tables

**Table 1:** Sample data from 22 locations of *Camponotus japonicus* used in this study.

Abbreviation	Location	Longitude	Latitude
BJHD	Haidian, Beijing	116°21′47.73″	39°58′31.44″
CQWL	Wulong, Chongqing	107°46′3.00″	29°19′55.56″
FJNA	Nanan, Fujian	118°27′35.64″	25°05′36.96″
GDRY	Ruyuan, Guangdong	113°14′13.92″	24°47′18.60″
GZGY	Guiyang, Guizhou	106°41′56.76″	26°36′16.92″
HBHD	Handan, Hebei	114°28′45.12″	36°37′48.72″
HBWH	Wuhan, Hubei	114°26′51.36″	30°33′52.56″
HLHE	Harbin, Heilongjiang	126°43′24.96″	45°43′30.36″
HNLY	Luoyang, Henan	112°27′18.86″	34°35′53.69″
HNSY	Shaoyang, Hunan	111°28′5.95″	27°12′26.84″
JSNJ	Nanjing, Jiangsu	118°51′35.28″	32°04′33.96″
JXNC	Nanchang, Jiangxi	115°45′56.52″	28°46′37.92″
LNSY	Shenyang, Liaoning	123°27′50.52″	41°50′41.64″
NMCF	Chifeng, Neimenggu	119°0′18.36″	42°17′56.04″
NMHH	Hohhot, Neimenggu	111°37′30.31″	40°52′17.73″
SCMY	Mianyang, Sichuan	104°43′35.40″	31°28′35.40″
SDTA	Taian, Shandong	117°09′57.26″	36°13′35.86″
SXJC	Jiaocheng, Shanxi	112°08′45.96″	37°34′40.08″
SXXX	Xixiang, Shannxi	107°45′58.32″	32°59′4.92″
SXYL	Yulin, Shannxi	109°43′15.75″	38°19′48.43″
XJAL	Altay, Xinjiang	88°07′21.62″	47°51′51.37″
ZJYY	Yuyao, Zhejiang	121°05′35.88″	29°43′50.52″

**Table 2:** Eigenvectors of the top 4 PCs in the rotated morphological analysis of major workers.

Variables	PC1	PC2	PC3	PC4
Body length	−0.443	−0.023	0.053	−0.272
Head length	−0.425	0.357	0.126	0.138
Head width	−0.426	0.359	0.071	0.192
Scape length	−0.188	−0.466	0.776	0.066
Pronotum width	−0.359	0.145	−0.075	0.470
Weber’s length	−0.370	−0.212	0.003	−0.571
Mandible length	−0.332	−0.208	−0.526	−0.235
Eye width	−0.172	−0.646	−0.301	0.513

**Table 3:** Eigenvectors of the top 4 PCs in the rotated morphological analysis of minor workers.

Variables	PC1	PC2	PC3	PC4
Body length	0.389	0.032	−0.216	0.398
Head length	0.415	−0.409	0.122	−0.204
Head width	0.421	−0.335	0.110	−0.379
Scape length	0.272	0.536	0.363	0.269
Pronotum width	0.429	−0.112	0.077	0.005
Weber’s length	0.358	0.227	0.340	0.210
Mandible length	0.198	0.607	−0.280	−0.683
Eye width	0.274	−0.001	−0.771	0.270

**Table 4:** Effects of climate zones, habitat types, and their interaction on the first 4 principal components (PC1–PC4) of major workers. Significant values are highlighted in bold.

	Explanatory Variables	Degrees of Freedom	Sum of Squares	H value	Original *p*-Value	Bonferroni- Corrected *p*-Value
PC1	**Climate zones**	**2**	**2,580,699**	**144.78**	**<0.001**	**<0.001**
	Habitat types	2	28,454	1.60	0.045	1
	**Climate zones × Habitat types**	**4**	**891,941**	**50.04**	**<0.001**	**<0.001**
	Residuals	453	4,555,362			
PC2	Climate zones	2	4161	0.23	0.015	1
	**Habitat types**	**2**	**553,548**	**31.05**	**<0.001**	**<0.001**
	**Climate zones × Habitat types**	**4**	**1,059,262**	**59.42**	**<0.001**	**<0.001**
	Residuals	453	6,576,795			
PC3	Climate zones	2	127,764	7.17	<0.001	0.333
	Habitat types	2	6972	0.39	0.031	1
	**Climate zones × Habitat types**	**4**	**780,054**	**43.76**	**<0.001**	**<0.001**
	Residuals	453	7,275,911			
PC4	Climate zones	2	170,646	9.57	0.0011	0.1
	Habitat types	2	313,024	17.56	<0.001	0.0018
	**Climate zones × Habitat types**	**4**	**643,018**	**36.07**	**<0.001**	**<0.001**
	Residuals	453	7,116,562			

**Table 5:** Effects of climate zones, habitat types, and their interaction on the first 4 principal components (PC1–PC4) of minor workers. Significant values are highlighted in bold.

	Explanatory Variables	Degrees of Freedom	Sum of Squares	H Value	Original *p*-Value	Bonferroni- Corrected *p*-Value
PC1	**Climate zones**	**2**	**533,849**	**29.95**	**<0.001**	**<0.001**
	Habitat types	2	14,133	0.79	0.673	1
	**Climate zones × Habitat types**	**4**	**1,194,862**	**67.03**	**<0.001**	**<0.001**
	Residuals	453	6,478,590			
PC2	**Climate zones**	**2**	**436,362**	**24.48**	**<0.001**	**<0.001**
	**Habitat types**	**2**	**921,037**	**51.67**	**<0.001**	**<0.001**
	**Climate zones × Habitat types**	**4**	**709,591**	**39.81**	**<0.001**	**<0.001**
	Residuals	453	6,200,106			
PC3	Climate zones	2	333,929	18.73	<0.001	0.0011
	Habitat types	2	228,464	12.82	0.002	0.020
	**Climate zones × Habitat types**	**4**	**1,660,191**	**93.14**	**<0.001**	**<0.001**
	Residuals	453	6,081,880			
PC4	**Climate zones**	**2**	**793,628**	**44.52**	**<0.001**	**<0.001**
	**Habitat types**	**2**	**815,734**	**45.76**	**<0.001**	**<0.001**
	**Climate zones × Habitat types**	**4**	**557,720**	**31.29**	**<0.001**	**<0.001**
	Residuals	453	6,192,454	29.95		

**Table 6:** Relationship between principal components (PC1–PC4) and environmental factors of major workers based on robust regression analysis.

	Independent Variable	Estimate	Std. Error	*t*-Value
PC1	Intercept	−3.4236	0.5091	−6.7245
	Annual mean temperature	0.113	0.0183	6.1702
	Annual mean precipitation	0.0175	0.0028	6.2281
	Relative humidity	−0.0025	0.009	−0.2737
	Elevation	0.0012	0.0002	6.6169
PC2	Intercept	1.0217	0.3749	2.7249
	Annual mean temperature	−0.0495	0.0135	−3.666
	Annual mean precipitation	0.0046	0.0021	2.2362
	Relative humidity	−0.0111	0.0066	−1.6787
	Elevation	−0.0001	0.0001	−0.3823
PC3	Intercept	1.0373	0.3164	3.2781
	Annual mean temperature	−0.0026	0.0114	−0.227
	Annual mean precipitation	0.0082	0.0017	4.721
	Relative humidity	−0.0223	0.0056	−3.9902
	Elevation	−0.0007	0.0001	−6.1976
PC4	Intercept	−1.4203	0.296	−4.7983
	Annual mean temperature	0.0106	0.0107	0.9957
	Annual mean precipitation	−0.0087	0.0016	−5.3408
	Relative humidity	0.0332	0.0052	6.3475
	Elevation	−0.0002	0.0001	−1.5807

**Table 7:** Relationship between principal components (PC1–PC4) and environmental factors of minor workers based on robust regression analysis.

	Independent Variable	Estimate	Std. Error	*t*-Value
PC1	Intercept	1.9129	0.3213	5.9534
	Annual mean temperature	−0.0418	0.0116	−3.6132
	Annual mean precipitation	0.0074	0.0018	4.1812
	Relative humidity	−0.0253	0.0057	−4.4553
	Elevation	−0.0008	0.0001	−6.8904
PC2	Intercept	1.9129	0.3213	5.9534
	Annual mean temperature	−0.0418	0.0116	−3.6132
	Annual mean precipitation	0.0074	0.0018	4.1812
	Relative humidity	−0.0253	0.0057	−4.4553
	Elevation	−0.0008	0.0001	−6.8904
PC3	Intercept	1.9129	0.3213	5.9534
	Annual mean temperature	−0.0418	0.0116	−3.6132
	Annual mean precipitation	0.0074	0.0018	4.1812
	Relative humidity	−0.0253	0.0057	−4.4553
	Elevation	−0.0008	0.0001	−6.8904
PC4	Intercept	1.1869	0.3072	3.8636
	Annual mean temperature	0.0263	0.0111	2.3809
	Annual mean precipitation	−0.0014	0.0017	−0.8359
	Relative humidity	−0.0188	0.0054	−3.461
	Elevation	−0.0004	0.0001	−4.086

## Data Availability

The original contributions presented in this study are included in Supplementary Materials.

## Supplementary Materials

The following supporting information can be downloaded at https://www.mdpi.com/article/10.3390/insects15090719/s1, Figure S1: Phylogenetic tree of *C. japonicus* inferred from Bayesian analysis of *COI* data; Table S1: Description of the ant traits examined in this study and their hypothesized functional response [74,75,76,77,78,79,80,81,82,83]; Table S2: Environmental factors of each sampling site.
