# Supplementary material for: Eco-Morphological Responses of Camponotus japonicus (Hymenoptera: Formicidae) to Varied Climates and Habitats
Source: Insects. 2024 Sep 19;15(9):719. doi: 10.3390/insects15090719 (PMC11431994; doi:10.3390/insects15090719)

Table S1. Description of the ant traits examined in this study and their hypothesized functional response. All measurements are in millimeters (mm). The illustrations of different parts of ant bodies were modified from Parr & al. (2017).

Trait	Measure	Hypothesized function	Source
Body length	Measured from tip of mandibles to tip of gaster, with the ant in an extended position	Size of gaps through which worker can pass; also linked to metabolic characteristics	Sarty et al., 2006
Head length	Maximum vertical length in full face view	Relates to diet, longer head length may indicate herbivory	Kaspari 1993; Sarty et al., 2006
Head width	Maximum horizontal length in full face view (across eyes)	Relates to mandible strength and predatory strategies	Kaspari 1993; Sarty et al., 2006
Eye width	Measured across the maximum width of the eye	Indicative of the exploratory capacities of the habitat	Grevé et al. 2019
Scape length	Maximum length of first antenna segment in a straight line	Relates to chemical sensory abilities. Longer scape facilitates pheromone trail following	Weiser & Kaspari 2006; Yates et al., 2014
Pronotum width	Pronotum longitudinal length in dorsal view	Indicative of body size and often linked with resource use and habitat maneuverability	Wiernasz and Cole 2003; Sarty et al. 2006
Weber's length	Maximum length measured from the anterior edge of pronotum to posterior edge of propodeum	Associated with food size, foraging preference and defense strategies	Weber, 1938; Kaspari & Weiser, 1999
Mandible length	Maximum straight-line from mandibular apex to anterior clypeal margin in full face view	Mandible size relates to predatory behavior, with larger mandibles allowing for larger prey capture	Weiser & Kaspari 2006; Gibb and Cunningham 2013

References for Tab. S1

Gibb, H.; Cunningham, S.A. Restoration of trophic structure in an assemblage of omnivores, considering a revegetation chronosequence. *J. Appl. Ecol.* **2013**, *50*, 449-458.

Kaspari, M. Body size and microclimate use in Neotropical granivorous ants. *Oecologia* **1993**, *96*, 500-507.

Kaspari, M.; Weiser, M.D. The size–grain hypothesis and interspecific scaling in ants. *Funct. Ecol.* **1999**, *13*, 530-538.

Parr, C.L.; Dunn, R.R.; Sanders, N.J.; et al. GlobalAnts: a new database on the geography of ant traits (Hymenoptera: Formicidae). *Insect Conserv. Divers*. **2017**, *10*, 5-20.

Sarty, M.; Abbott, K.L.; Lester, P.J. Habitat complexity facilitates coexistence in a tropical ant community. *Oecologia* **2006**, *14*, 465-473.

Weber N.A. The biology of the fungus-growing ants. Part VII. The Barro Colorado Island, Canal Zone, species. *Rev. Entomol*. **1941**. *12*, 93-130.

Weiser, M.D.; Kaspari, M. Ecological morphospace of New World ants. *Ecol. Entomol.* **2006**, *31*, 131-142.

Wiernasz, D.C.; Cole, B.J. Queen size mediates queen survival and colony fitness in harvester ants. *Evolution* **2003**, *57*, 2179-2183.

Yates, M.L.; Andrew, N.R.; Binns, M.; Gibb, H. Morphological traits: predictable responses to macrohabitats across a 300 km scale. *PeerJ* **2014**, *2*, e271.

Grevé, M.E.; Bláha, S.; Teuber, J.; Rothmaier, M.; Feldhaar, H. The effect of ground surface rugosity on ant running speed is species-specific rather than size dependent. *Insectes Soc.* **2019**, *66*, 355-364.

Table S2. Environmental factors of each sampling site.

Abbreviation	Annual mean temperature (°C)	Annual mean precipitation (mm)	Relative humidity (%)	Elevation (m)
BJHD	13.18	64.00	53.07	52
CQWL	14.55	121.51	80.12	264
FJNA	21.52	125.13	75.56	35
GDRY	20.34	123.99	74.98	525
GZGY	14.42	106.11	83.14	1088
HBHD	16.22	80.09	51.06	98
HBWH	18.43	115.63	70.80	42
HLHE	4.75	47.82	74.14	141
HNLY	16.58	80.36	56.94	142
HNSY	18.47	121.33	75.22	239
JSNJ	16.98	114.17	72.82	106
JXNC	18.02	159.11	73.73	316
LNSY	8.74	73.43	68.20	46
NMCF	7.84	37.16	53.71	224
NMHH	8.27	32.71	50.22	1180
SCMY	17.84	96.99	68.57	529
SDTA	11.59	136.63	64.30	207
SXJC	12.39	39.61	50.55	756
SXXX	12.39	39.61	50.55	436
SXYL	10.18	32.75	48.37	1086
XJAL	-0.65	17.85	51.74	1275
ZJYY	15.07	190.53	78.21	759

Note: Data is obtained from NOAA-National Centers for Environmental Information.

Figure S1. Phylogenetic tree of *C. japonicus* inferred from Bayesian analysis of *COI* data.

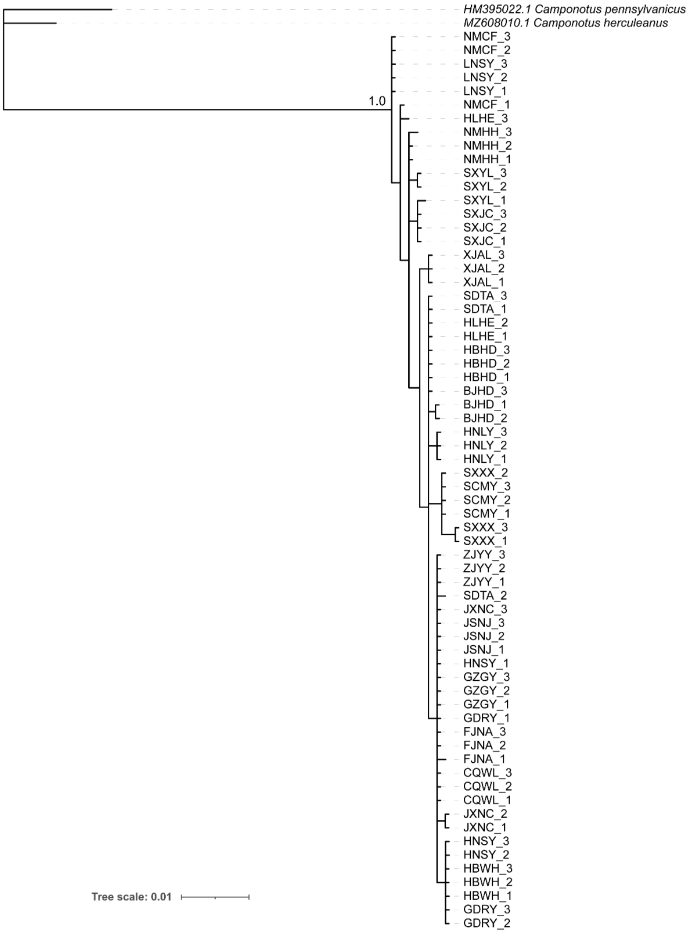

Supplement: Supplementary file 1 [file insects-15-00719-s001.zip › insects-3177094-supplementary.pdf]
